# Editorial: Advances in drug discovery for anti-Alzheimer’s agents

**DOI:** 10.3389/fchem.2022.1028829

**Published:** 2022-09-23

**Authors:** Lhassane Ismaili

**Affiliations:** Université Bourgogne Franche-Comté, Besançon, France

**Keywords:** MTDL, alzheimer’s disease, multifunctional agents, multifactorial disease, neurodegenerative disease

Alzheimer’s disease (AD) is a progressive and fatal neurodegenerative disorder, known to be the main cause of dementia in elderly populations (Dementia, 2022). Several factors such as intracellular formation of neurofibrillary tangles (NFTs) and neuropil threads (NTs) resulting from abnormal hyperphosphorylation of tau protein, the aggregation and extracellular deposition of amyloid beta (Aβ) protein, metal-ion dysregulation which is known to effect the Aβ peptide—trigging its aggregation and increasing toxicity, and reduction of acetylcholine (ACh) levels in the brain are responsible for the onset and progression of AD. Clinical trials achieved based on the single-target approach have not been successful, indicating that treatment of AD needs multi-targeting agents. (Ismaili et al., 2022).

Despite the tremendous efforts and financial expenditures aimed at finding an efficient treatment for AD, no definitive cure has been found so far. As AD is a multi-factorial disease, single-targeted drugs usually do not work effectively. As a result, design and development of synthetic compounds or natural products possessing multi-target anti-AD activity are in high demand. Recently, a wide range of *in vitro* and *in vivo* tests against amyloid beta (βA) and tau protein aggregation have been reported in the literature. Aducanumab, an amyloid beta-directed monoclonal antibody, has been recently approved by FDA (Research, C. for D. E, 2021). Additionally, inhibition of beta-secretase 1 (BACE1) and cholinesterases (ChEs), as well as development of biometals chelators and inflammatory drugs have been found to be important in the treatment of AD.

In this special issue of Frontiers in Chemistry, a series of colleagues from different research laboratories present their ideas and latest work on the development of anti-Alzheimer’s agents, which we hope will attract the attention and interest of the reader.

Certainly, multi-targeted molecules for AD have concentrated most of the research papers presented here and submitted by the Pourabdi et al., Hu et al., Babaei et al., and Ciaramelli et al. The Mahdavi et al. contributed with a review article on benzylpyridinium salts: mimicking donepezil in the treatment of AD.

Thus, the Mahdavi et al., updates the state of the art in the design of MTDL-based benzyl-pyridinium salts as a scaffold mimicking donepezil. The authors discussed, based on SAR studies, the most potent substituents and moieties that are responsible for inducing the desired activity. The goal is to understand the potential of this biologically scaffold for Alzheimer’s disease drug discovery.

Pourabdi et al. described the synthesis of new series of N-benzyl triazole-linked coumarin derivatives, and evaluated them against 15-lipoxygenase (15-LOX), and acetyl- and butyrylcholinesterase (AChE and BuChE). Among the synthesized compounds, the authors identified two promising compounds as potential MTDLs for further investigation against AD, showing BuChE and 15-LOX inhibition activities as well as significant neuroprotective and anti-amyloid aggregation activities.

Ciaramelli et al., by combining molecular recognition studies based on NMR, atomic force microscopy, and *in vitro* biochemical and cellular assays, investigated the anti-amyloidogenic properties of cinnamon extracts. From this work, the authors identified flavanols, particularly procyanidins and cinnamaldehydes, as ligands and inhibitors of Aβ_1-42_ aggregation. Along with the previously reported ability to prevent tau aggregation, these results indicate that cinnamon polyphenols are natural and promising scaffolds for developing multi-targeted anti-AD compounds.

Babaei et al. describes a novel serie of coumarin-based scaffolds linked to pyridinium salts via flexible aliphatic carbon chains, and their evaluation as MTDLs against AD. Very interestingly, the identified promising compounds showed strong nanomolar inhibition against AChE and BuChE with ability reduce β-amyloid self and AChE-induced aggregation and significantly protect PC12 and SH-SY5Y cells against H_2_O_2_-induced cell death and amyloid toxicity.

These results suggest that the new designed hybrids of coumarin and pyridinium parts could be considered as promising multifunctional agents for further developments in the field of anti-Alzheimer drugs.

Finally, Hu et al. investigate the potential of α- Mangostin (α-M) as anti-AD agent as well as its structure-activity relationship by comparing the differences between α-M and several analogues from Xanthone family. All the compounds studied showed a significant ability to inhibit Aβ fibrillogenesis and resulted in a decrease in Aβ_1-42_ and inflammatory cytokine levels. Some may also enhance neuronal viability against Aβ-induced neurotoxicity and preserved the integrity of the bEnd.3 tight junction against LPS-induced neuroinflammation.

Structure-activity analysis identified several groups and modifications essential for the bioactivity of these xanthones.

In summary, the growing interest in the development of MTDLs, targeting several sites of action, is an interesting and perfectly adapted way to find an efficient therapeutic solution for complex and multifactorial neurodegenerative diseases, in particular AD. This special issue is a contribution to this field.
